# Paediatric cataract: challenges and complications

**Published:** 2016

**Authors:** P Vijayalakshmi, Lucy Njambi

**Affiliations:** Chief: Paediatric Ophthalmology & Strabismus Department, Aravind Eye Hospital, Madurai, India.; Lecturer and paediatric ophthalmologist: University of Nairobi, Nairobi, Kenya.

**Figure:**
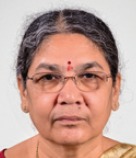
Dr P Vijayalakshmi

**Figure:**
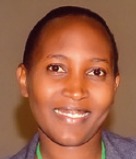
Lucy Njambi

## Detection and management of amblyopia

Amblyopia should always be anticipated in children with unilateral cataract, asymmetrical bilateral cataracts (or where there is a delay between the first and second eye operation, or a delay of more than a year between diagnosis/ detection and surgery), cataracts with anisometropia or traumatic cataracts with corneal scars. When amblyopia is detected, occlusion therapy (eye patching) must be instituted at the earliest opportunity. The patching regimen is the same with any strabismic amblyopia and sometimes needs to be aggressive at the start. It is crucial to explain the need for patching to the parents, since compliance is the greatest obstacle to the success of amblyopia treatment.

## Myopic shift

As all children are prone to a myopic shift, the axial length should be measured at every visit. A more rapid shift is seen in those operated early in life with emmetropic correction in infancy. Frequent refraction is necessary for optimal optical correction. Children under the age of 8 years undergoing IOL surgery should be slightly under-corrected, leaving them slightly hyperopic so that they can grow into emmetropia, thereby preventing very high myopia later.

## Management of low vision

Even with uncomplicated cataract surgery and a clear visual axis, some children still end up with low vision due to amblyopia or other ocular or central nervous system abnormalities such as cerebral palsy, periventricular leucoma-lacia, congenital rubella syndrome, etc. These children should be referred for vision rehabilitation.

**Figure:**
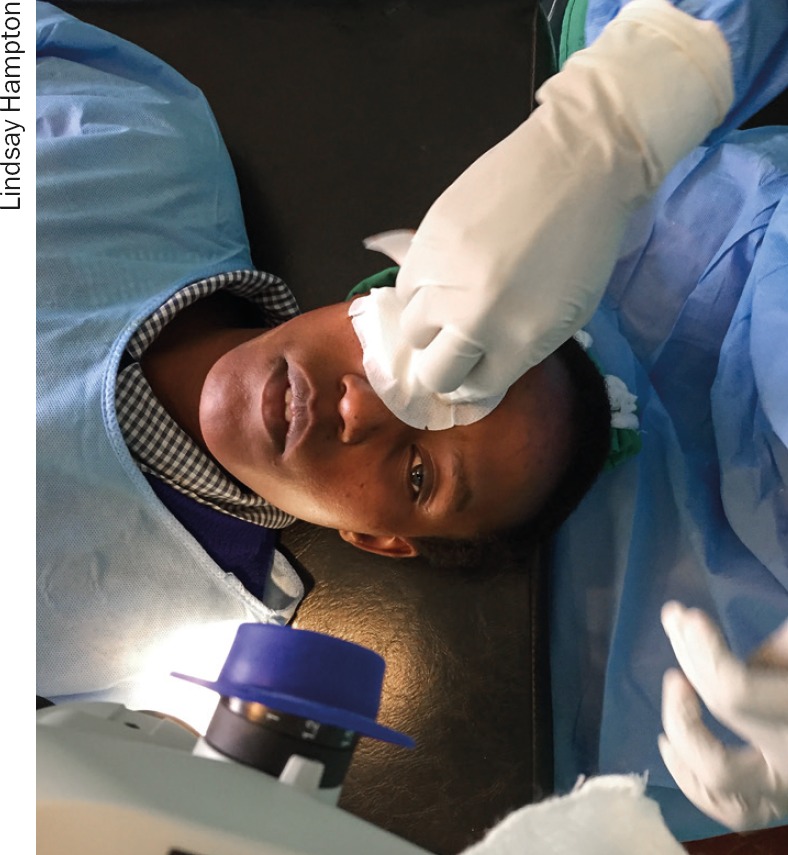
Applying an eye pad after cataract surgery. KENYA

## Secondary IOL

A decision about whether to insert a secondary IOL in aphakic patients should be considered with caution, taking into account the status of eyeball growth (especially the anterior segment), glaucoma, posterior capsule support, and the potential to improve visual acuity. The sulcus is the easiest position for secondary lens implantation, although this has a higher risk of decentration. With the availability of improved technology and various lenses, each child who is in need should be given a choice, provided the parents understand the visual prognosis.

## Timing of the second eye operation

In infants under the age of 12 months with bilateral cataracts, the risk of amblyopia is very high after non-IOL surgery. Surgery on the second eye is recommended 2 to 3 days after the first eye during the same admission (total admission 5-6 days). Where anesthesia risk is high, both the eyes can be operated in a single sitting.

If the parents of children undergoing IOL surgery are poorly resourced or have travelled a long way and may not come back for an operation on the second eye, then this can also be done a few days later, as most inflammation is seen within the first few days after surgery.

Bilateral surgery is becoming increasingly common in some countries, particularly in centres with limited access to a paediatric anaesthesiologist and when parents may not return for surgery on the second eye. Another consideration is the lower risk of repeated general anaesthesia. Strict aseptic measures must be observed to reduce the risk of bilateral endophthalmitis. Each eye is treated as a separate procedure with repeat scrubbing, gowning and gloving of the surgeon and assistant. A new sterile instrument set must be used for the second eye. Contraindications include upper respiratory and ocular infections, congenital nasolacrimal duct obstruction and children at risk of increased inflammation such as those with juvenile rheumatoid arthritis.

## Complications

### Visual axis opacification

Visual axis opacification and membrane formation is common, particularly in young children. For significant opacity, i.e. with reduced visual acuity or where fundus details cannot be seen, YAG capsulotomy can be tried. Surgical membranectomy is required if YAG is not available or fails, or if a soft after-cataract (secondary cataract) has developed. This is best avoided by doing a primary posterior capsulotomy and anterior vitrectomy up until the age of 6-9 years. In older children, a prophylactic Nd:YAG laser capsulotomy can be done at the one week or one month follow-up, when the posterior capsule is unlikely to be fibrosed.

**‘Glaucoma is common in children after surgery for congenital cataract’**

### Glaucoma

Glaucoma is common in children after surgery for congenital cataract and is difficult to manage. It is more frequent with microphthalmos, microconea, congenital rubella syndrome, anterior segment anomaly (such as aniridia, ectopia lentis, orspherophakia) and in traumatic cataract and those operated for cataract in infancy. It can occur many years after the operation. IOP measurement and recording is therefore mandatory at all visits and central corneal thickness should be measured where indicated. Anti-glaucoma medication should be prescribed after consultation with a glaucoma expert. Apart from a rise in intraocular pressure, other important signs of glaucoma are an increase in axial length, rapid loss of hypermetropia or an increase in myopia and optic disc cupping.

### Postoperative uveitis

The incidence of severe postoperative uveitis has reduced with better surgical techniques, modern IOLs, in-the-bag placement of IOL, and less manipulation of the iris. Heparin-coated IOLs or intracameral heparin, where available, can also reduce the risk of uveitis. Early and frequent use of topical, peri-ocular and systemic steroids in some cases can usually control the inflammation. The trick is to ensure an in-the-bag placement of the IOL to minimise IOL and iris touch and subsequent iris chafing.

### Retinal detachment

Although retinal detachment is rare the retina should be examined at each visit particularly in eyes with long axial lengths or where surgery was complicated. Retinal examination can be challenging due to small pupils and peripheral capsular opacities.

### Endophthalmitis

Treatment for endophthalmitis in children is in principle the same as for adults. After surgery, loose sutures should be removed as they predispose to infection.

**Figure:**
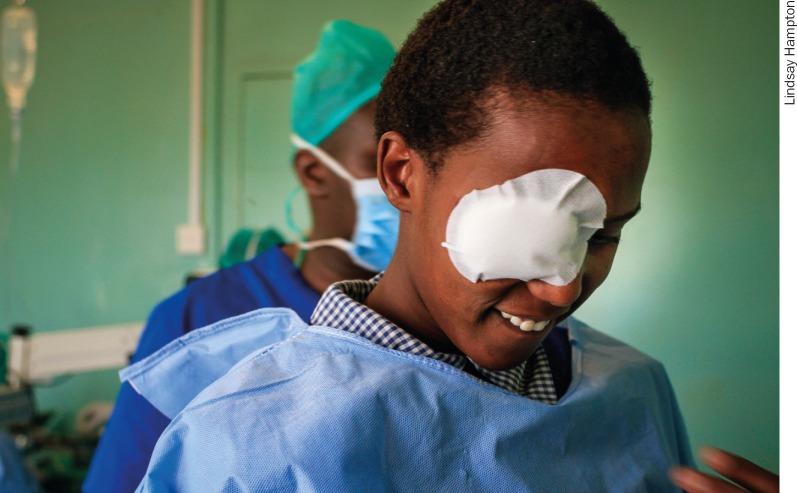
A 13-year-old girl after cataract surgery. Good postoperative care is essential in order to avoid complications. KENYA

